# Maintaining prevention focus: the impact of an interdisciplinary taskforce on CAUTI rates during an EMR transition

**DOI:** 10.1017/ash.2024.411

**Published:** 2024-10-25

**Authors:** Rachel Pryor, Le Kang, Shelley Knowlson, Amy Britton, Christoph Lecznar, Michelle Doll, Georgia McIntosh, Gonzalo Bearman

**Affiliations:** 1 Virginia Commonwealth University Health System, Richmond, VA, USA; 2 Department of Biostatistics, Virginia Commonwealth University, Richmond, VA, USA; 3 Richmond VA Hospital, Richmond, VA, USA

## Abstract

We employed an interrupted time series analysis to assess the impact of changing electronic medical records, concurrent with a Catheter-Associated Urinary Tract Infection (CAUTI) Taskforce, on CAUTI rates. We found that rates increased in most ICUs before returning to baseline. These findings suggest that a multi-faceted approach may reduce CAUTI rates.

## Introduction

Catheter-associated urinary tract infections (CAUTI) are one of the most frequent healthcare-associated infections, representing 44% of all diagnosed urinary tract infections.^1,2^ An estimated 12%–16% of acute and critical care patients will undergo indwelling urinary catheterization, which increases the potential for CAUTI.^1^ CAUTIs are associated with an increased cost to the healthcare system at an estimated range of $603–1764 per event and are associated with an increase in length of stay and mortality.^1^

Studies show that electronic medical record (EMR)-based tools may help to reduce CAUTI rates,^3,4^ From May 2015 to January 2021 the Healthcare Infection Prevention Program (HIPP) at Virginia Commonwealth University Medical Center (VCUMC) worked with our Information Technology team to embed multiple EMR decision support tools, best practice alerts, and hard stops to aid in decreasing CAUTI rates in our legacy EMR. Our facility announced its plan to discontinue our legacy EMR (where these EMR prompts were built) and transition to a new EMR in 2020.

An interdisciplinary CAUTI Taskforce was formed in March 2021. Hospital leaders requested the Taskforce identify system barriers associated with noncompliance with CAUTI best practice standards and establish accountability standards that uphold urinary catheter (UC) best practice. The Taskforce highlighted order entry issues to be addressed during the new EMR build. While the CAUTI taskforce was designed to impact the entire organization, work began in the adult ICUs as pilot units and then systematically rolled out to non-ICU areas. The Taskforce included key stakeholders representing HIPP, Performance Improvement, Nursing Leadership, and Ordering Practitioners.

Due to successful CAUTI decreases in our Surgical Trauma Intensive Care Unit (STICU) and Neuroscience ICU (NSICU), the Taskforce based its educational resources and nurse leader escalation documents on documents first shared in those units. The Taskforce shared these resources and documents with all other adult ICUs in September 2021. The Taskforce also escalated its concerns about CAUTI rates with hospital executives using a Situation, Background, Assessment, Recommendation (SBAR) document in September 2021.

In December 2021, VCUMC transitioned EMRs, leading to a temporary loss of most of the previous automated interventions that existed in the legacy EMR. A urinalysis (UA)/reflex order entry with required testing indication was built into the new EMR at go-live, but the orders for UC insertion, maintenance, and removal all fired at the same time, leading to confusion for nursing staff. The CAUTI Taskforce continued its work throughout the EMR transition period. In September 2022, the Taskforce delivered organization-wide safety topic announcements during the Daily Safety and Operations Briefing. Topics included appropriate testing criteria, locations of educational resources, and messaging encouraging interprofessional engagement among team members. We seek to determine whether the EMR transition that occurred during the ongoing work of the Taskforce impacted CAUTI rates.

## Methods

We conducted retrospective interrupted time series (ITS) analyses between September 2019 and December 2022 based on CAUTI incidence data for different ICUs. Our goals were to model the time series pattern before specific interventions versus the pattern after the interventions, and to statistically assess if the time series pattern had changed significantly in relation to the preintervention pattern. Specifically, we estimated the level change and trend change in standardized monthly CAUTI infection rates prior to the first intervention, as well as the changes in level and trend following the first intervention and subsequent interventions. The level change 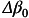 is defined as the estimated 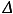 between two adjacent time points immediately before and after the intervention, and the trend change 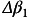 is defined as the estimated 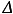 between two slopes (rates of change) based on time series data before and after the intervention.

The interventions of interest were (1) Nurse leader escalation and education, (2) executive leadership SBAR, (3) implementation of a new EMR, (4) organizational weekly safety topic announcement. The primary outcomes were standardized CAUTI rate defined as monthly CAUTI count/1000 catheter days and test normalized CAUTI rate which is defined as the standardized CAUTI incidence rate divided by test rate in each ICU unit. All analyses were performed using SAS® v9.4 (SAS Institute, Cary, NC, USA) with a significance level of 0.05 unless otherwise specified. No multiplicity adjustments were performed. The UC Standardized Utilization Ratios (SUR) for all units were relatively stable throughout the ITS analysis time period, so we opted to exclude the SURs in the final analysis.

## Results (Table 1, Figure 1)

### Medical respiratory ICU (MRICU)

We found a significant level increase ( 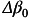 = 0.136, *P* = 0.036) after nurse leader escalation and education/executive leadership SBAR followed by a significant trend decrease ( 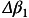 = –0.063, *P* = 0.029) in test normalized CAUTI rate. We also found a significant level increase in test normalized CAUTI rate after discontinuation of legacy EMR ( 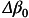 = 0.094, *P* = 0.048).

**Table 1.** Statistically significant level change or trend change for study outcomes

Standardized CAUTI rate			
	Intervention	Level change (Δ 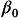 )	Trend change
NSICU	A	–0.404 (*P* = 0.793)	–0.655 (*P* = 0.006)
STICU	A	–2.606 (*P* = 0.033)	–0.355 (*P* = 0.049)
Test normalized CAUTI rate			
Burn ICU	C	0.531 (*P* = 0.026)	–0.075 (*P* = 0.576)
MI-ICU	C	0.129 (*P* = 0.046)	–0.018 (*P* = 0.595)
MRICU	A/B	0.136 (*P* = 0.036)	–0.063 (*P* = 0.029)
	C	0.094 (*P* = 0.048)	0.055 (*P* = 0.059)
NSICU	A	0.062 (*P* = 0.135)	–0.012 (*P* = 0.042)
STICU	C	0.463 (*P* = 0.012)	0.022 (*P* = 0.837)

Intervention List:

A. Education & Nurse Leader Escalation.

B. Executive Leadership SBAR.

C. EMR Transition from Cerner to Epic.

D. Organizational Weekly Safety Topic Announcement (because we analyzed only 3 months of data after the organizational weekly Safety Topic Announcement, the ITS estimates for this specific intervention are not reliable and none are significant).

**Figure 1.**
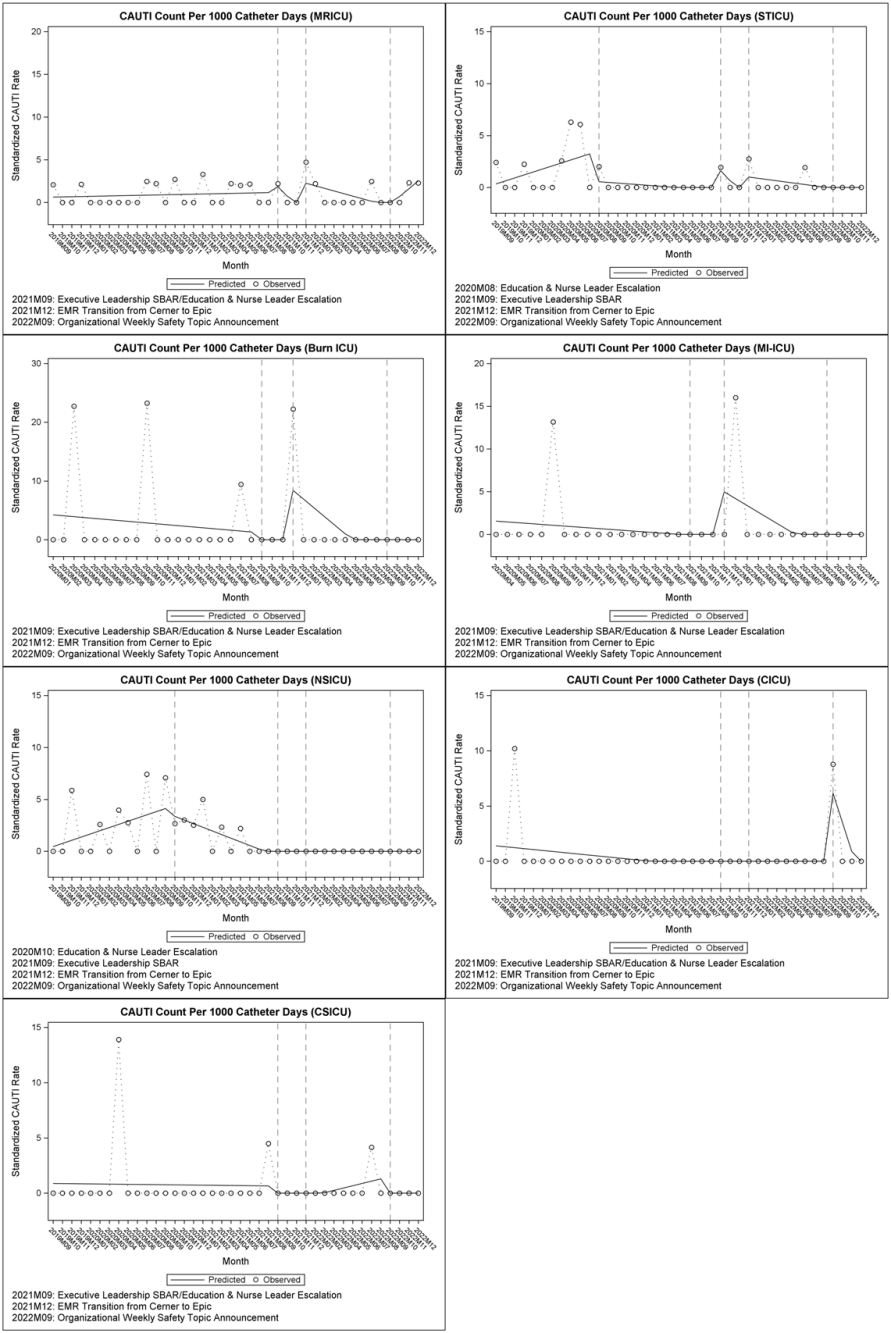
Interrupted time series outcomes.

### Surgical trauma ICU (STICU)

We found a significant level decrease ( 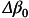 = –2.606, *P* = 0.033) followed by a significant trend decrease ( 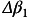 = –0.355, *P* = 0.049) after nurse leader escalation and education in standardized CAUTI rate. We found a significant level increase in test normalized CAUTI rate after discontinuation of legacy EMR ( 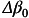 = 0.463, *P* = 0.012).

### Burn ICU (B-ICU), medical intermediate and intensive care unit (MI-ICU)

We found a significant one-time level increase in test normalized CAUTI rate after discontinuation of the legacy EMR for Burn ICU ( 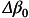 = 0.531, *P* = 0.026) and MI-ICU ( 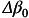 = 0.129, *P* = 0.046).

### Neuroscience ICU (NSICU)

We found a significant trend decrease ( 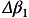 = –0.655, *P* = 0.006) in standardized CAUTI rate after nurse leader escalation and education. There was a significant trend decrease ( 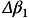 = –0.012, *P* = 0.042) in test normalized CAUTI rate after nurse leader escalation and education.

### Cardiac ICU (CICU), cardiac surgery ICU (CSICU)

We did not find any intervention statistically significant in either unit.

## Discussion

We assessed the impact of an EMR transition on CAUTI rates while the interventions by the CAUTI Taskforce endured. We hypothesized that the change of EMR-based triggers would adversely impact CAUTI rates. With a change in the EMR, some of the previously built prompts for UC discontinuation were not included.

We did not see evidence of sustained negative impact on CAUTI rates following a change in EMR. The reasons for this are multiple and may include the work of the Taskforce to empower nursing unit-level leadership and focused attention towards provider-nurse collaboration. Despite the change in the EMR at our institution, our study describes how education and leadership engagement, along with new EMR prompts, may result in a sustained reduction in CAUTI.

Study strengths include the use of standardized CAUTI definitions as published by the Centers for Disease Control National Healthcare Safety Network. Data was collected by trained HIPP professionals. We employed an ITS analysis to best measure a change in the slope of the rate of CAUTIs. Study weaknesses include a single-center design, with results that may not be generalized. Due to the fact, we analyzed only three months of data after the organizational weekly Safety Topic Announcement, the ITS estimates for this specific intervention are not reliable and none are significant.

We report no significant impact of a change in EMR on institutional, global CAUTI rates. These findings may reflect the multi-faceted approach employed for CAUTI prevention, one which relies heavily on urinary catheter insertion and maintenance education along with a nurse-driven de-escalation algorithm that encourages interprofessional collaborative practices. Future studies are required to better understand the proportionate impact of leveraging the EMR to reduce urinary catheter use and minimize CAUTI risk.
